# Comparison of Wet Fractionation Methods for Processing Broccoli Agricultural Wastes and Evaluation of the Nutri-Chemical Values of Obtained Products

**DOI:** 10.3390/foods11162418

**Published:** 2022-08-11

**Authors:** Éva Domokos-Szabolcsy, Nevien Elhawat, Geraldo Jorge Domingos, Zoltán Kovács, Judit Koroknai, Enikő Bodó, Miklós Gábor Fári, Tarek Alshaal, Nóra Bákonyi

**Affiliations:** 1 Department of Applied Plant Biology, Institute of Crop Sciences, University of Debrecen, Böszörményi Street 138, 4032 Debrecen, Hungary; 2 Department of Biological and Environmental Sciences, Faculty of Home Economic, Al-Azhar University, Tanta 31732, Egypt; 3 Agricultural and Molecular Research and Service Institute, University of Nyíregyháza, 4407 Nyíregyháza, Hungary; 4 Soil and Water Department, Faculty of Agriculture, Kafrelsheikh University, Kafr El-Sheikh 33516, Egypt

**Keywords:** broccoli, agro-waste, microwave coagulation, lacto-fermentation, leaf protein concentrate, brown juice, protein, phytochemicals

## Abstract

The main objective of this study was to increase the economic value of broccoli green agro-waste using three wet fractionation methods in the shadow of green biorefinery and the circular economy. Product candidates were obtained directly by using a mechanical press, and indirectly by using microwave coagulation or via lactic acid fermentation of green juice. The leaf protein concentrates (LPC) fractions displayed significantly higher dry matter content and crude protein content (34–39 m/m% on average) than the green juice fraction (27.4 m/m% on average), without considerable changes in the amino acids composition ratio. UHPLC-ESI-ORBITRAP-MS/MS analysis showed that kaemferol and quercetin are the most abundant flavonols, forming complexes with glycosides and hydroxycinnamic acids in green juice. Lacto-ermentation induced a considerable increase in the quantity of quercetin (48.75 μg·g^−1^ dry weight) and kaempferol aglycons (895.26 μg·g^−1^ dry weight) of LPC. In contrast, chlorogenic acid isomers and sulforaphane disappeared from LPC after lactic acid fermentation, while microwave treatment did not cause significant differences. These results confirm that both microwave treatment and lacto-fermentation coagulate and concentrate most of the soluble proteins. Also, these two processes affect the amount of valuable phytochemicals differently, so it should be considered when setting the goals.

## 1. Introduction

Due to the growing demand for protein, the European Union (EU) agricultural sector has had to pay special attention to ensuring adequate quality and quantity of protein in the food chain. Plants are among the protein sources of particular importance. Today’s food and feed industry are built mainly on seed-based protein sources, including cereals and legumes (mainly soybean). However, most EU countries are not self-sufficient in soybean, and they have to import soybean from third countries. However, imported soy is constantly exposed to fluctuations in market prices, affecting profits in the food production chain. Therefore, finding alternative sources of protein is of vital interest.

Regarding the protein distribution within plant organs in addition to seeds, protein accumulates in high concentrations in the leaves. The protein composition of green leaves is more heterogeneous than the stored protein in the seed endosperm or cotyledon. Along with it, the most abundant leaf protein is the RuBisCo enzyme (ribulose-1,5-bisphosphate carboxylase/Oxygenase enzyme), which can constitute 30–50% of the soluble cell protein in C3 plants [1]. It is located in the stroma of the chloroplast.

The philosophy of green biorefining builds on the protein and other valuable biomolecules in green biomass/leaves to create value-added products for food, feed or other industrial purposes with minimal waste emission. Green biorefining can involve both dedicated biomass plant species such as alfalfa and annual/perennial grasses or green residues of plant crops [2].

Broccoli (*Brassica oleracea* L. var. Italica) is a member of the *Brassicaceae* family, which includes a variety of vegetables that are consumed worldwide, such as cabbage, cauliflower, brussels sprouts, and radishes [3]. Due to their health promoting effects, the consumption of cruciferous foods has increased across Europe in recent years. At the same time, broccoli’s harvest produces a massive amount of agro-wastes, particularly large-sized leaves. Broccoli is one of the crops with only 10–15% of the plant’s total aerial edible biomass [4]. A considerable amount of the plant residue is abandoned in the field or at the processing facility during the harvesting, sorting, and processing. According to Liu et al. [5] after harvesting the heads/florets, the leaves, stalks, and stems (about 70% of the plant) are left on the fields and plowed back into the soil as green fertilizer. This waste of precious resources results in the loss of nutritious green biomass, as well as investments in limited resources like water, fertilizer, farmland, and energy, all of which contribute to greenhouse gas emissions [6].

Broccoli heads have long been recognized as a vital ingredient of a balanced, healthy diet. They have a low-calorie content of 34 kcal (142 kJ)/100 g FW, and are a good source of minerals (i.e., calcium, phosphorus, potassium, and sodium), vitamins (i.e., B, C, E, and K), fibers, amino acids and a variety of other health-promoting molecules, such as carotenoids (ß-carotene and lutein), flavonoids (kaempferol), hydroxycinnamic acids (sinapic and caffeoyl-quinic acid derivatives), and, most importantly, glucosinolates (GLs) [7,8]. At the same time, the leaf also contains valuable phytonutrients. Prade [9] reported that between 34–42 kg of total protein could be extracted per one ton of dry matter of broccoli leaves. Liu et al. [5] cited the significantly higher accumulation of carotenoids (carotene, violaxanthin, neoxanthin, and lutein) chlorophylls, vitamins E and K in leaves than in florets. Also, higher phenolic acids contents, including chlorogenic acids, sinapic acid, caffeic acid, and ferulic acid were detected in microwave-assisted broccoli leaf extracts than in florets [10]. Phytocompounds in broccoli organs such as sulphoraphane showed anti-inflammatory properties in vitro and in animal studies [11].

Despite its valuable substances, the direct eating of broccoli leaves is not attractive to consumers. Different extraction processes of green biomass/waste can contribute to provide products with concentrated proteins and other desirable functional properties [12]. Generally, the first step of wet processes is a mechanical disruption to separate the soluble proteins from the fibrous parts. The proteins are then concentrated in simple step or combination of steps by using thermal coagulation or by the use of acidic/alkaline precipitation or flocculants [13]. By-products originated from broccoli’s leaves for food and feed purposes are available with information about their protein and phenolic compounds contents [9].

In agreement with these purposes, the current study aimed to valorize the neglected green biomass (mainly leaves) of broccoli, comparing three wet fractionation methods, including a novel microwave-assisted coagulation, lactic acid fermentation, and direct mechanical pressing. The protein quantity and quality of the obtained fractions were evaluated in the context of the applied processes. Furthermore, the quantitative changes in the phytochemicals of the obtained fractions were documented.

## 2. Materials and Methods

### 2.1. Chemicals and Reagents

For the chemical analysis, HCl (analytical reagent grade) (a.r.), 37%, NaOH (≥97.0%) LC–MS grade water, and a 3kDa PES membrane filter were ordered from VWR International, Radnor, USA. The AccQ-Tag Ultra derivatization reagent kit, a mixture of standard proteinogenic amino acids, the AccQ-tag Ultra eluent A and AccQ-tag Ultra eluent B were purchased from Waters (Waters, Milford, MA, USA). The gradient grade methanol (MetOH) (≥99.9%) and standards of phytochemical compos: nicotinamide (≥98%), nicotinic acid (a. s.), biotin (≥99%), riboflavin (a. s.), liquiritigenin (≥97% (HPLC), formononetin (a. s.), chlorogenic acid (analytical standard), neochlorogenic acid (≥98%), chryptochlorogenic acid (a. s.), syringaldehyde (98%), sulforaphane (≥90% synthetic, liquid), sinapic acid (≥98%), scopoletin (analytical standard), apigenin (≥95.0%); apigenin-7-O-glucuronide (primary reference standard); luteolin (≥98%); quercetin (≥95.0%); isoquercitrin (a. s.); naringenin (≥95.0%); genkwanin (≥98%); kaempferol (≥97.0% (HPLC); isoliquiritigenin (a. s.), and ferulic acid (USP reference standard) were obtained from Sigma-Aldrich (Darmstadt, Germany).

### 2.2. Plant Source

Broccoli (*Brassica oleracea* var. Italica) seedlings were cultivated in an open field at the Demonstration garden of the University of Debrecen, Hungary (47°32′0”; 21°38′0” E) in 2020. The lant-to-plant distance was 40 × 60 cm and plants were irrigated individually by using the PoliDrip Standard (Poliext Ltd., Kecskemét, Hungary) drip irrigation system (consisting of 20 mm ⌀ tubes) delivering water directly to the root zone of the plant. The experimental plots did not receive fertilizers or pesticides before or during the growing period of broccoli plants. Adapted to broccoli growing practices, the leafy shoots were collected when the florets reached marketable size.

### 2.3. Processing of Green Biomass

**Figure 1:**
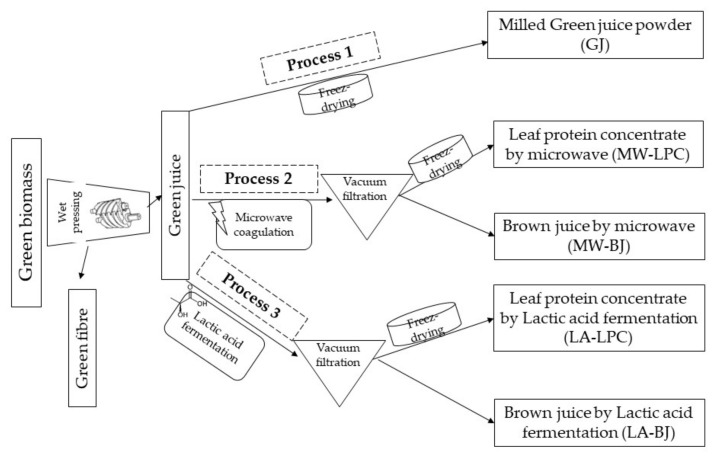
Schematic chart flow of the three applied protein extraction processes of broccoli green biomass, including the obtained product candidates/fractions.

Different wet processing methods of broccoli fresh green leaves were compared to obtain fractions as summarized in Figure 1. These three processes started with a common wet fractionation step using the Angel Juicer twin screw (5500, Angel Ltd., Anyang, South Korea), which resulted in mechanically pressed fiber and green juice (GJ) fractions. According to *Process 1,* GJ was directly freeze-dried by an Alpha 1-4 LSC plus-Martin Christ (Germany) device and then powdered by a stainless steel grinder. The *Process 2* was based on an alternative thermal coagulation method using microwave radiation as described by Fári and Domokos-Szabolcsy [14], where the GJ was coagulated in one step at 80 ± 2 °C using an intermittent microwave device (Samsung M1711N, South Korea) set for 450 Watt. The coagulated “green cottage cheese” was further separated by vacuum filtration using a filter membrane with a pore size of 5 µm. The *Process 2* resulted in a solid protein rich leaf protein concentrate (MW-LPC) and liquid brown juice (MW-BJ) fractions (Figure 1). The MW-LPC was then freeze-dried and powdered, as mentioned in Process 1. The MW-BJ was kept at −20 °C for further biochemical analysis.

In *Process 3*, the protein isolation was based on acidic precipitation using lacto-fermentation of the GJ. The lacto-fermentation was induced by applying 1 M lactic acid at a rate of 5% to the fresh GJ, and then incubated for four days at 36 °C under anaerobic conditions. The coagulated “green cottage cheese” was further separated by vacuum filtration as mentioned in *Process 2,* yielding solid protein rich leaf protein concentrate (LA-LPC) and liquid brown juice (LA-BJ) fractions. The LA-LPC was freeze-dried and powdered as mentioned in Processes 1 and 2, while the LA-BJ was stored at −20 °C.

### 2.4. Physicochemical Parameters of Obtained Products

Dry matter content of the freeze-dried GJ, MW-LPC, and LA-LPC fractions was calculated based on the fresh and dry masses. The pH and water-soluble dry material content Brix (°Bx) of the MW-BJ and LA-BJ fractions were measured before freeze-drying using a pH meter (Mettler Toledo S20 Seven Easy, Switzerland) and refractometer (RBR32-ATC manual refractometer, Polling, Germany), respectively.

### 2.5. Determination of Crude Protein Content

The crude protein content of the GJ, MW-LPC, MW-BJ, LA-LPC, and LA-BJ fractions was measured as total N content using the Kjeldahl method according to the ISO 5983-2:2009 international standard method. The crude protein content was calculated by using a nitrogen conversion factor of 5.6 [15].

### 2.6. Quantification of Amino Acids Composition

The amino acid composition of GJ, MW-LPC, MW-BJ, LA-LPC, and LA-BJ fractions was measured by ultrahigh-pressure liquid chromatography (UHPLC) using a Waters Acquity H-class plus UPLC System (Waters, Milford, MA, USA). Briefly, a 20 mg sample was placed in 50 mL-digestion tubes and hydrolyzed with 6 M HCl in a microwave digestion unit (CEM MARS One, Matthews, NC, USA). The pH of the hydrolyzed acidic sample was adjusted with 6M NaOH. The sample was filtered by a 3kDa PES membrane filter (VWR International, Radnor, PA, USA).

The separation was based on AccQTag pre-column derivatization chemistry. According to the manufacturer’s instructions, the hydrolyzed and neutralized samples were derivatized using an AccQ-Tag Ultra derivatization reagent kit. The sample derivatization process was the same as was used in the case of standard amino acid mixtures [16].

The separation of the derivatized amino acids was carried out on an AccQ-tag Ultra C18 column (1.7 µm; 2.1 × 100 mm, Waters, Millford, MA, USA) guarded by an Accquity in-line filter (0.2 µm; 2.1 mm, Waters, Millford, MA, USA). An 11 min long gradient elution was set up with 0.100 mL min^−1^ flow rate. The column temperature was 54 °C. A Quaternary gradient pump mixed solvent A, which was 100% AccQ-tag Ultra eluent A, while solvent B was 10% AccQ-tag Ultra eluent B in LC–MS grade water, solvent C was LC water, and solvent D was 100% AccQ-tag Ultra eluent B. The results were evaluated with Waters Empower 3 software (Waters, Millford, MA, USA).

### 2.7. Protein Expression Pattern by SDS-PAGE

The soluble protein expression pattern of broccoli fractions was evaluated by sodium dodecyl sulfate–polyacrylamide gel electrophoresis (1D SDS-PAGE). Ten mg of freeze-dried sample or 400 µL of liquid sample were quantified and mixed with an 800 uL solubilization buffer (2× Laemmli). After vigorous vortexing, the samples were incubated for 5 min at 95 °C, followed by 15 min centrifugation at 13,000 rpm at 4 °C. The supernatant was used for further analysis. The SDS-PAGE was performed by a vertical system on a discontinuous polyacrylamide gel. The resolving gel was 12.5%; and the stacking gel was 9%, prepared in a Mini-Protean Tetra Cell gel system (Bio-Rad Inc. Hercules, MI, USA). The gels were stained with Coomassie G250 staining solution and were then analyzed using the BioRad ChemiDoc MP Imaging System.

### 2.8. Qualitative and Quantitative Phytochemical Analysis

Freeze-dried fractions, i.e., GJ, MW-LPC, and LA-LPC, were extracted by methanol:water (70:30 ratio) with continuous shaking at 150 rpm for 2 h at room temperature in the dark. The extracts were then filtered using a PTFE filter with a pore size of 0.22 µm. The liquid MW-BJ and LA-BJ fractions were filtered directly by a PTFE filter. Qualitative analyses of phytochemicals were performed from the GJ fraction by the UHPLC-ESI-ORBITRAP-MS/MS hyphenated analytical system as described in our prior work [17]. For chromatography, a Dionex Ultimate 3000RS UHPLC system (Thermo Fisher, Waltham, MA, USA) was served with a Thermo Accucore C18 analytical column (2.1 mm × 100 mm, 2.6 µm particle size). Gradient elution was performed at a 0.2 mL min^−1^ flow rate with methanol and water eluents. Samples were measured in positive and negative ionization modes. Each chemical compound was processed according to retention time, molecular weight, and ion fragmentation pattern using Thermo Trace Finder 2.1 software. For identification, self-collected and online databases were used. The processed data were manually checked using Thermo Xcalibur 4.0 software.

Considering the results of the qualitative analysis, quantification of some selected components was determined using the same UHPLC-MS system. For the quantification, an external calibration curve was generated. Quantitative determination was made taking into account the following standard compounds: nicotinamide (≥98%), nicotinic acid (a. s), biotin (≥99%), riboflavin (a. s), liquiritigenin (≥97% (HPLC), formononetin (a. s), chlorogenic acid (analytical standard), neochlorogenic acid (≥98%), chryptochlorogenic acid (a. s), syringaldehyde (98%), sulforaphane (≥90% synthetic, liquid), sinapic acid (≥98%), scopoletin (analytical standard), apigenin (≥95.0%); apigenin-7-O-glucuronide (primary reference standard); luteolin (≥98%); quercetin (≥95.0%); isoquercitrin (a. s); naringenin (≥95.0%); genkwanin (≥98%); kaempferol (≥97.0% (HPLC); isoliquiritigenin (a. s.), and ferulic acid (USP reference standard). A 30 min long gradient was established. Gradient elution was made with MetOH and water (buffer A, water; buffer B, MetOH delivered at 0. 2 mL min^−1^ flow rate. The program was as follows: 0–2 min, 95% A and 5% B; 2–20 min, up to 100% B; 20–22 min, 100% B; 22–23 min, down to 5% B; 23–30 min, 95% A and 5%B.

### 2.9. Statistical Evaluation

Normality and homoscedasticity of the dependent variables were checked and transformed as necessary. Data analysis was performed using Microsoft Excel 2016 and the SPSS 25.0 software package (SPSS Inc., Chicago, IL, USA). The analysis of variance using one-way ANOVA was performed between processed fractions. Separation of means was performed by post-hoc test (Tukey’s test), and significant differences were accepted at the level of *p* ≤ 0.05. The data were presented as mean ± standard deviation.

## 3. Results

### 3.1. Yield and Physicochemical Traits of Processed Products of Broccoli Green Leaves

Three wet fractionation methods were compared for the production of value-added products by harvesting the green residue of broccoli after the economically mature florets had been harvested. The applied methods are summarized in Figure 1 in the Materials and Methods section.

#### 3.1.1. Fresh Yield

**Figure 2:**
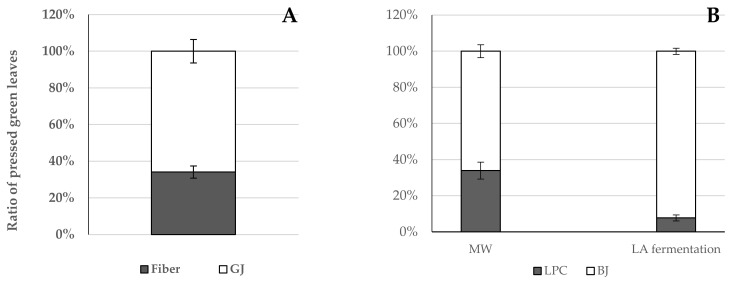
Percentage distribution of wet fractions from the processed broccoli green biomass: (**A**): Fibre and green juice fractions resulting from wet pressing; (**B**): Percentage of leaf protein concentrate and brown juice fractions from broccoli green juice resulting from microwave coagulation (MW) or lactic acid fermentation (LA). Data are presented as mean ± SD (*n* = 3).

The yield of the processed products obtained from broccoli fresh green leaves by different fractionation methods is presented in Figure 2. The results revealed that the GJ and fiber fractions represented 65.9% and 34.1%, of the mechanically pressed fresh green leaves of broccoli (Figure 2A), respectively. The BJ fraction was almost twofold that of the LPC fraction, regardless of the extraction method. The isolation of leaf protein via the microwave coagulation method displayed markedly higher LPC content (35.3%) than the lacto-fermentation method (~13%) (Figure 2B).

#### 3.1.2. Dry Matter Content of Processed Products of Broccoli Green Leaves

**Figure 3:**
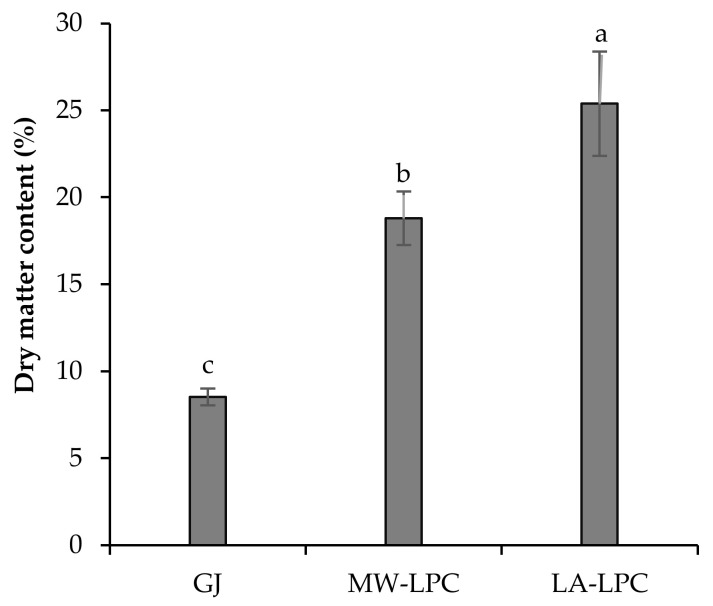
Dry matter content (%) of the freeze-dried fractions of the processed broccoli fresh green leaves. GJ is obtained after the mechanical pressing of the green leaves, the MW-LPC is the leaf protein concentrate obtained by the thermal coagulation of the fresh GJ using a microwave device, and LA-LPC is the leaf protein concentrate obtained by the lacto-fermentation process of the fresh GJ. Different letters on the columns show significant differences according to Tukey’s test at the level of *p* ≤ 0.05. Data are presented as mean ±SD (*n* = 3).

Dry matter content was calculated based on the fresh and dry masses of the freeze-dried products, i.e., GJ, MW-LPC, and LA-LPC (Figure 3). The different fractions showed significantly different dry matter contents. The GJ fraction exhibited the lowest dry matter content at 8.53%, while the MW-LPC and LA-LPC fractions showed dry matter content of 18.80% and 25.39%, respectively. Results confirmed that the method applied to isolate LPC is of importance, as the lacto-fermentation method resulted in a higher content of LPC than the microwave coagulation method.

#### 3.1.3. pH and Brix of GJ and BJ Fractions

**Figure 4:**
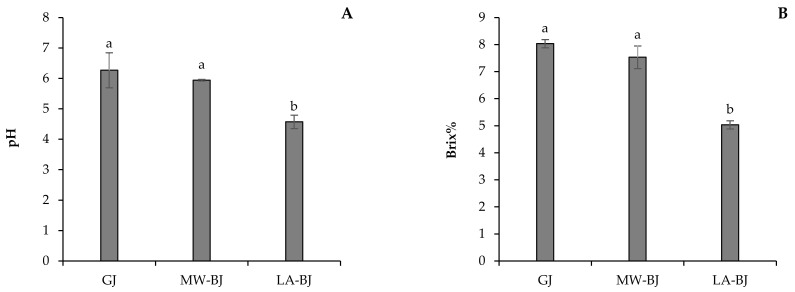
(**A**) pH and (**B**) Brix value (%) of the liquid fractions obtained after the processing of the broccoli fresh green leaves by different fractionation methods. GJ is the fresh green liquid obtained after the mechanical pressing of the fresh green leaves of broccoli, MW-BJ is the brown liquid obtained after the thermal treatment of the fresh GJ to isolate the leaf protein using a microwave device, and LA-BJ is the brown liquid obtained after the lacto-fermentation of the fresh GJ to isolate the leaf protein. Different letters on the columns show significant differences according to Tukey’s test at the level of *p* ≤ 0.05. Data are presented as mean ± SD (*n* = 3).

The liquid fractions, i.e., GJ, MW-BJ, and LA-BJ, of the processed broccoli green leaves displayed significantly different pH and Brix values (Figure 4A,B). The fresh GJ fraction showed the highest pH (6.27), whereas the lacto-fermentation process of the fresh GJ resulted in the lowest pH (4.57). The results indicated that the lacto-fermentation process was effective in reducing the pH of the BJ fraction (Figure 4A). This is an advantage for the storage of brown juice, as the fresh BJ fraction is unstable at room temperature and spoils quickly. However, lowering the pH of the BJ increases its stability and means that it can be stored for a longer period. Along with pH, the lacto-fermented BJ fraction displayed the lowest Brix value. Both GJ and MW-BJ fractions exhibited similar Brix values of 8.03 and 7.53, respectively (Figure 4B).

### 3.2. Biochemical Assessment of Processed Broccoli Green Leaves

#### 3.2.1. Crude Protein Content and Amino Acids Composition

The protein content of freeze-dried green juice was 27.43 m/m% as measured by the Kjeldhal method (Table 1). Appling coagulation processes to the GJ fraction obtained by the mechanical pressing of broccoli fresh biomass significantly increased the crude protein content in LPC and BJ fractions compared to the freeze-dried GJ fraction (Table 1). Microwave coagulation increased the crude protein content of the MW-LPC fraction by ~25%, resulting in a 34.30 m/m% protein content compared to freeze-dried GJ (27.43 m/m%). The lacto-fermentation process also resulted in an increase of 43% of crude protein content in the LA-LPC compared to the freeze-dried GJ. The BJ fraction displayed significantly lower crude protein content compared to the LPC fraction. The microwave coagulation process exhibited higher protein content (1.96 m/m%) in BJ fraction compared to the BJ obtained through the lacto-fermentation process, which resulted in a protein content of 1.30 m/m%. Regarding the amino acid composition, most of the proteinogenic amino acids in the BJ fractions were below the detection limit. Aspartic and glutamic acids were the most abundant amino acids, regardless of the fraction. Among the nutritionally essential amino acids, lysine and methionine displayed the highest concentration (1.403 and 0.455 g 100 g^−1^ DW, respectively) in the MW-LPC. In contrast, arginine, as a conditionally essential amino acid, was detected in the highest concentration (2.371 g 100 g^−1^ DW) in the LA-LPC.

#### 3.2.2. Protein Expression Pattern

**Figure 5:**
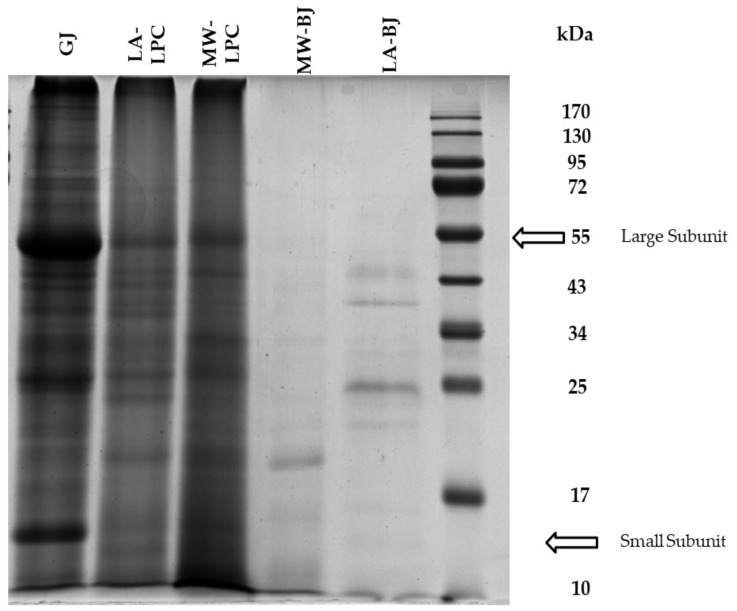
SDS-PAGE analysis of broccoli green leaves-derived fractions by different processes (*Process 1; Process 2 and Process 3*): freeze-dried green juice (GJ); leaf protein concentrate obtained by microwave coagulation (MW-LPC); leaf protein concentrate obtained by lactic acid fermentation (LA-LPC); brown juice obtained by microwave coagulation (MW-BJ); and brown juice obtained by lacto-fermentation (LA-BJ).

Changes in protein expression patterns of fractions obtained from broccoli green leaves are depicted in Figure 5. The GJ, as a result of Process 1, showed the highest number of bands due to the high amount of soluble proteins. The MW-LPC contained the least number of bands compared to the LA-LPC and GJ fractions. However, there are some common bands appearing in LPCs and GJs. These include the large subunit of Rubisco at ~55 kDa and the small subunit of Rubisco at ~14 kDa. The microwave coagulation and lacto-fermentation methods showed very few protein bands. Faint bands with a weak appearance were noticed in the BJs, particularly the MW-BJ.

#### 3.2.3. Screening of Phytochemicals of GJ

The phytochemical composition of broccoli green leaves-derived GJ was revealed by the UHPLC-ESI-ORBITRAP-MS/MS. The GJ is the end product of process 1, and at the same time it is the platform product of process 2 and 3. As Table 2 shows, seven water-soluble vitamins were identified. Among the phytochemicals, flavonoids were the highest identified in the GJ fraction. In agreement with the literature, quercetin (3,3′,4′,5,7-Pentahydroxyflavone), kaempferol (3,4′,5,7-Tetrahydroxyflavone) aglycones and their various derivatives belonging to the flavonols were the most abundant while isorhamnetin (3,5,7,4-tetrahydroxy-3 -methoxyflavone) derivatives were identified at lower numbers [18]. Most of the kaempferol and quercetin form complexes with acylated di-, tri- or tetra-glycosides and hydroxycinnamic acids, such as caffeic, ferulic, and sinapic acids, since they are frequent in the side chain [19]. Some flavones, flavanones and chalcone, such as apigenin (4′,5,7-Trihydroxyflavone), apigenin-7-O-glucuronide and luteolin (3′.4′.5.7-Tetrahydroxyflavone) were also detected. Broccoli GJ is also a rich source of phenolic acids. All the three isomers of caffeoylquinic acid were identified, i.e., chlorogenic acid (3-O-Caffeoylquinic acid), neochlorogenic acid (5-O-Caffeoylquinic acid) and chrytochlorogenic acid (4-O-Caffeoylquinic acid), with a characteristic [M + H]^+^ ion at *m*/*z* 355.103 (Table 2). Moreover, sinapic acid and its glycosyl derivatives, such as di-O-sinapoylglucose were also found in the broccoli GJ. Among glucosinolates, glucobrassicin and neoglucobrassicin as indole glucosinolate quantified with the sulforaphane.

#### 3.2.4. Quantitative Analysis of the Phytochemicals of Fractions Obtained by Different Processes

The quantitative alterations of some health-relevant phyto-components concerning the fractionation methods were investigated in the GJ, MW-LPC, LA-LPC, MW-BJ, and LA-BJ products. The results showed that nicotinic acid is the most abundant water-soluble vitamin. Significantly, higher values were measured in the GJ (19.18 μg g^−1^ DW) than in the MW-LPC and LA-LPC (11.97 and 11.33 μg g^−1^ DW), respectively (Table 3). The highest nicotinamide content (9.71 μg g^−1^ DW), also known as vitamin B3, was found in the green juice fraction. Protein coagulation processes reduced its concentration significantly. Regarding the BJs, nicotinic acid significantly reduced by the lacto-fermentation process (1620.00 ng mL^−1^) compared to the microwave coagulation process (2093.35 ng mL^−1^). Biotin was only detectable in the GJ in small amounts, while it was below the detection limit in both the LPC and BJ fractions, regardless of the extraction method.

Among the quantified flavonoids, quercetin (3,3′,4′,5,7-Pentahydroxyflavone) and kaempferol (3,4′,5,7-tetrahydroxyflavone) were the most abundant in all the fractions, regardless of the extraction method (Table 4). The microwave coagulation method resulted in a significant increase (~10-fold) in kaempferol and quercetin aglycones contents in the MW-LPC compared to the GJ. However, as a result of lacto-fermentation, the concentrations of both quercetin and kaempferol aglycones in the LA-LPC increased by at least one order of magnitude compared to the GJ. For instance, the concentration of kaempferol was 2.63 μg g^−1^ DW in the GJ and increased to 895.26 μg g^−1^ DW in the LA-LPC. Likewise, a significant increase in the content of kaempferol and quercetin aglycones were observed in the LA-BJ compared to the MW-BJ. For example, as shown in Table 4, the concentration of quercetin was 209.66 ng mL^−1^ in the MW-BJ compared to 7390 ng mL^−1^ in the LA-BJ. Isoliquitigenin (2′,4,4′-trihydroxychalcone), belonging to the chalcones, liquitirigenin (4′.7-Dihydroxyflavanone) and naringenin (4′,5,7-Trihydroxyflavanone), belonging to the flavanones, and genkwanin, luteolin (3′,4′,5,7-Tetrahydroxyflavone), apigenin (4′,5,7-Trihydroxyflavone) and apigenin-7-O-glucoronide, belonging to the flavones, were detected as minor phytocompounds. The concentrations of these flavonoids ranged between 0.11 to 3.13 μg g^−1^ DW in the GJ, MW-LPC, and LA-LPC fractions, and from 1.09 to 80.34 ng mL^−1^ in the MW-BJ and LA-BJ, respectively. However, in some cases, the concentrations of these components were below the detection limit.

Among phenolic compounds, 12 phytochemical components were qualitatively identified in the GJ (Table 2). The three isomers of the chlorogenic acid were detected and they were the most abundant. Among them, neochlorogenic acid (5-O-Caffeoylquinic acid) was the dominant isomer, typically accumulating in different fractions in an order of magnitude higher than cryptochlorogenic acid (4-O-Caffeoylquinic acid) and chlorogenic acid (3-O-Caffeoylquinic acid) (Table 5). It is striking that the lacto-fermentation process reduced the concentration of all the three chlorogenic acid isomers, while they were below the detection limit in the LA-LPC. A similar dramatic decrease was noticed in the LA-BJ compared to the MW-BJ. The concentration of sinapic acid was 352.20 μg g^−1^ DW in the GJ. Its concentration decreased to 208.51 μg g^−1^ DW in the LPC after the microwave coagulation (MW-LPC). On the other hand, the lacto-fermentation process resulted in a larger decrease, recording 109 μg g^−1^ DW of in the LA-LPC. Sulforphane, as a glucosinolate compound characteristic of broccoli, was detected at a concentration of 17.65 ng in the GJ, while it was almost absent in the LA-LPC and LA-BJ, possibly due to the lacto-fermentation process.

## 4. Discussion

The present study investigates the valorization potential of broccoli green leaves as side streams through a comparative evaluation of three processes in the green biorefineries. Wet fractionation is the first step of the general green conveyor belt, which results in GJ and lignocellulosic fiber fractions. Although the GJ of broccoli at pH 6.27 contains valuable carbohydrates (Brix% is 8.03) and proteins, it is perishable in this form. For this reason, it is necessary to convert the GJ into a storable form, preferably increasing its nutritional value. The simplest solution is to reduce the water content of the GJ by freeze-drying. The GJ, as a product of Process 1, displayed a dry matter content of 8.53%. Thermal coagulation, including heat exchangers or direct steam injection, is widely used to precipitate proteins from green juices [13,20,21]. In our work, thermal coagulation was achieved by microwave radiation as described in a recent patent by Fári and Domokos-Szabolcsy [14]. Microwave coagulation resulted in protein coagulates with a compact structure here referred to as MW-LPC, which was easily separated from a liquid portion called MW-BJ. This method resulted in significant changes in the quality of the obtained MW-LPC compared to the freeze-dried GJ obtained through Process 1. Similar to Process 1, the final step of Process 2 is freeze-drying of the MW-LPC to avoid microbial spoilage; the dry matter content of the MW-LPC increased by 18.80%. The protein content also showed a significant increase with a result of 34.40 m/m%. The third alternative method to preserve the broccoli GJ together with proteins recovery was the lacto-fermentation as presented in Process 3 (Figure 1). Acidification of the GJ with organic acids and/or lactic acid bacteria is a well-known option in green biorefining with several advantages [22]. A decrease in pH leads to the precipitation of soluble proteins in the GJ, which can be concentrated by filtration into a value-added protein product candidate (LA-LPC) and LA-BJ. In this work, lactic acid and the inherent microorganisms in the GJ of broccoli were used to lower the pH. Lactic acid was added to facilitate the start of fermentation. By vacuum filtration, about ~13% of the fermented “green cottage” was collected as protein concentrate (LA-LPC). Comparing the two coagulation methods, the microwave process resulted in considerably higher LPC yields than lactic acid fermentation (Figure 2). By contrast, the freeze-dried LA-LPC showed the highest significant dry matter content (25.39%) compared to the GJ and MW-LPC (Figure 3), as well as displaying high protein content (39.18 m/m%). Another advantage of lacto-fermentation of the fresh GJ is the reduction in pH value that can inhibit the growth of acid sensitive antagonistic pathogens. The proliferation of lactic acid bacteria (LAB) has an added value when LA- LPC is used as feed, as LAB is part of the natural flora of the gastrointestinal tract of animals and humans, which is involved in the probiotic effects and the detoxification of carcinogens [23]. The brown juice obtained as a by-product of lactic acid fermentation showed a markedly higher amount than the result of microwave coagulation. In detail, 70% liquid brown juices were left over from the microwave treated green cottage, while more than 80% of the LA-BJ was generated by lacto-fermentation (Figure 2). Not only the quantity but also the quality of the BJ was affected by the processing method of the fresh GJ, recording significant differences in the composition of MW-BJ and LA-BJ. For instance, the Brix value of the LA-BJ dropped significantly to 5.03; this may be ascribed to converting most of the carbohydrates in the GJ organic acids, mainly lactic acid [24]. The crude protein content of the LA-BJ was also lower; however, the summarized amino acids were higher than that of the MW-BJ. The applied microwave and lacto-fermentation processes concentrated the proteins into a solid fraction, which was confirmed by the increased crude protein content. The summarized values of amino acids composition showed a similar tendency to the crude protein content, but lower values were reported. Accordingly, higher total amino acids contents were measured in the MW-LPC and LA-LPC than in the GJ. At the same time, functional properties of proteins considerably changed. While proteins in the GJ were typically in soluble form, heating provoked the proteins coagulation due to the opening of hydrophobic sites and the denaturation of proteins [25] and lacto-fermentation led to precipitation of several soluble proteins. Most bands on the SDS-PAGE were detected in the GJ fraction, confirming the presence of a large number of soluble proteins (Figure 5). On the contrary, few bands were detected with less intensity for the MW-LPC, despite its significantly high crude protein content. This could be attributed to the fact that the MW-LPC is largely composed of coagulated protein aggregates, which could be solubilized to a small extent. Typically, imperfectly coagulated copies of some abundantly expressed proteins such as Rubisco subunits are seen with weak appearance [1]. Even fewer and weaker bands could be detected in the BJs than in the LPCs due to the vacuum filtration, which retains the precipitated/coagulated proteins in solid phase. The measured crude protein content (1.30–1.96 m/m%) may be derived from minor peptides, amino acids [26] and other N-compounds.

Green biomass contains valuable phytochemicals in addition to proteins, in varying proportions and composition, depending on plant species. Although phytochemicals do not have an essential nutritional value, they possess a number of health-promoting properties depending on their biological activity [5,27,28]. Among others, clinical trials indicate that quercetin, as one of the most ubiquitous polyphenols, has beneficial effects on cardiovascular diseases and inflammation. Along with this, it possesses a higher bioavailability and thus bioactivity in the form of glucoside conjugates, than aglycone. Furthermore, the flavonoid isoquercitrin (quercetin-3-O-β-D-glucopyranoside) shows a chemopreventive impact against oxidative stress, cancer and allergic reactions [29]. Among the phenolic acids, the therapeutic role of chlorogenic acid is the most well-known. The antiobesity and antidiabetic properties of chlorogenic acid are associated with glucose metabolism [30].

The health benefits and other biological activities of the *Brassica* species may be attributed, on one side, to a diverse group of polyphenols, including flavonoids and phenolic acids [31]. For instance, a positive correlation was found between total phenolic compounds in broccoli green leaf extract and the inhibition of cancer cell growth [5]. Anti-inflammatory and chromatin modifying impacts of glucoraphanin derivatives of cruciferous has also been provided in in vitro and animal studies [11].

Here, qualitative analysis of phytochemicals of broccoli green leaves-derived GJ was carried out by the UHPLC-ESI-MS in both negative and positive ESI modes. Quercetin, kaemferol and isorhamnetin and their derivatives, belonging to the flavonols, were the most abundant flavonoids (Table 2). In agreement with Cartea et al. [32], quercetin, kaempferol and isorhamnetin were revealed, especially as O-glycosides. For instance, 21 identified kaempferol glycosides/hexosides were detected in the broccoli GJ. The number of sugar moieties were elevated and most of them were acylated by various hydroxycinnamic acids such as caffeic acid or sinapic acid. The quantitative changes of quercetin and kaempferol aglycones in solid fractions (i.e., GJ, MW-LPC, and LA-LPC) were also monitored, where results showed an extraordinary increase in the processed LPCs compared to the GJ (Table 4). According to Podsedek [31], food processing may induce the conversion of flavonoid aglycones from conjugated flavonoids. Microwave coagulation could be interpreted as a form of food/feed processing, producing a ~10-fold increase in the MW-LPC. Lacto-fermentation was even more pronounced than microwave treatment, resulting in a ~340-fold increase in kaemferol aglycon content (895.26 μg g^−1^ DW). This is probably due to the fact that the lactic acid bacteria, in addition to the lacto-fermentation treatment, used the glycosyl moieties of the conjugated kaemferol derivatives, resulting in a relative increase in the concentration of aglycones. Miean et al. [33] reported that the kaemferol content of broccoli’s edible part was below the detection limits. However, another study [28] documented the existence of kaempferol in broccoli at a concentration of 7.20 mg kg^−1^ FW. The kaempferol and quercetin concentrations of BJs were similar to those reported in the LPCs. For example, LA-BJ displayed ~13.047 ng mL^−1^ kaempferol, while MW-BJ showed only 340 ng mL^−1^. Hence BJ could modulate important physiological routes in plants, therefore it can be used as plant conditioner/biostimulant [34]. Among other flavonoids, relatively few numbers of chalcones, flavones and flavonones were presented in broccoli green leaves-derived GJ. These minor components did not show outstanding differences in concentration compared to the GJ and LPCs, although statistically verifiable differences were observed in most cases.

In addition to flavonoids, phenolic acids were also predominant. Out of these, chlorogenic acids were formed by esterification of hydroxycinnamic acid derivatives with quinic acid. In agreement with the literature, neochlorogenic acid was the main isoform of caffeoylquinic acid in the processed broccoli fractions [35]. However, the maximum concentration in broccoli sprout juice was 12.03 μg mL^−1^, while our experiment showed that the concentration of neochlorogenic acid in the MW-BJ was 94.88 μg mL^−1^ (Table 5). After neochlorogenic acid, cryptochlorogenic acid (4-O-Caffeoylquinic acid) was in the second highest concentration. The isomeric distribution of caffeoylquinic acids typically varies between plant families. While neochlorogenic acid was the predominant isoform in broccoli green leaves-derived products, chlorogenic acid was the most abundant isoform in Jerusalem artichoke green biomass-derived LPC [17]. In contrast to the findings of the amount of chlorogenic acid isomers in the different broccoli green leaves-derived products, kaempferol and quercetin aglycones exhibited an opposite tendency. Almost the same concentrations of chlorogenic acids were measured in the GJ and MW-LPC, while in the LA-LPC they were below detection limits. In BJs, the concentration of the three chlorogenic acid isomers also dropped as a result of the lacto-fermentation process. Among the glucosinolates, the quantitative changes of sulforaphane were monitored because of its biological activity and health protective effects. It is converted from biologically inert glucoraphanin to sulforaphane by the enzyme myrosinase as a result of physical/mechanical effects [35,36,37]. The first step in the processing of broccoli green leaves was the mechanical pressing that may induce the bioconversion of glucoraphanin to sulforaphan. The myrosinase enzyme released from previously intact cell organelles liberating the sulforahpane. This unstable aglycone was most abundant in the freshly pressed and untreated GJ (17.65 μg g^−1^ DW). Due to its instability, significantly lower amounts of sulforaphane were detected in the MW-LPC that were subjected to heat treatment. The lacto-fermentation had an even more negative effect, as sulforaphane was below the detection limit in both LA-LPC and LA-BJ.

## 5. Conclusions

In the present work, the green juice (GJ) obtained through the mechanical pressing of broccoli leaves was treated in different ways, including freeze-drying, microwave coagulation, and lacto-fermentation, obtaining concentrated leaf protein (LPC). A by-product, referred to as brown juice, was also obtained via microwave coagulation and lacto-fermentation. Microwave coagulation of freshly pressed GJ beneficially increased the dry matter and the crude protein content of the MW-LPC compared to the direct freeze-dried GJ. However, from the two protein concentration methods, lacto-fermentation increased the crude protein content of the LPC fraction more but with lower yields, in addition to altering the amino acid composition. Furthermore, the lacto-fermentation process has the advantage of reducing the pH of the by-product brown juice, which accounts for 60% of the green biomass, and thus increasing its shelf-life. To our knowledge, this study is the first that compares microwave coagulation (as an alternative method of thermal coagulation) with the lacto-fermentation process to isolate leaf protein concentrate and brown juice from fractionated broccoli green biomass.

As concerns the phytochemical composition, it largely depended on the applied protein isolation method. The results showed a marked increase in the amount of flavonol aglycones, especially quercetin and kaempferol, during the lacto-fermentation process compared to microwave coagulation. However, the contents of some vitamins B, including nicotinamide, nicotinic acid, biotin, and riboflavin, were higher in the freeze-dried GJ, without any further processing. Regarding the quantitative changes of phenolic acids, especially chlorogenic acid isomers, p-coumaric acid, and ferulic acid, it is clearly preferable to either coagulate the fresh GJ by microwave technique or to freeze-dry the fresh GJ.

Overall, both methods can effectively concentrate proteins dissolved in green juice. However, considering the ratio and the phytochemical composition of the fractions obtained, the wet processes have both advantages and disadvantages compared to each other, which should be taken into account when scaling up.

## Figures and Tables

**Table 1:** Crude protein content and amino acid composition of broccoli green leaves-derived fractions by different processes (*Process 1; Process2 and Process 3*): freeze-dried green juice (GJ); leaf protein concentrate obtained by microwave coagulation (MW-LPC); leaf protein concentrate obtained by lactic acid fermentation (LA-LPC); brown juice obtained by microwave coagulation (MW-BJ); and brown juice obtained by lacto-fermentation (LA-BJ). Data are means ± SD (*n* = 2 (for amino acids) and 3 (for crude protein content)). One-way ANOVA performed for the crude protein content of all the fractions (*p* ≤ 0.05).

	GJ	MW-LPC	LA-LPC	MW-BJ	LA-BJ
	Crude Protein Content (m/m%)				
	27.43 ± 0.12 c	34.30 ± 1.77 b	39.18 ± 0.17 a	1.96 ± 0.01 a	1.30 ± 0.03 b
	Amino acids composition				
	g 100 g^−1^ DW			g 100 g^−1^ FW	
His	0.286 ± 0.12	0.503 ± 0.01	0.253 ± 0.01	0.001 ± 0.00	0.002 ± 0.00
Asn	0.000 ± 0.00	0.000 ± 0.00	0.000 ± 0.00	0.000 ± 0.00	0.001 ± 0.00
Ser	1.285 ± 0.49	1.217 ± 0.01	1.673 ± 0.48	0.002 ± 0.00	0.007 ± 0.00
Gln	0.000 ± 0.00	0.000 ± 0.00	0.000 ± 0.00	0.000 ± 0.00	0.000 ± 0.00
Arg	1.194 ± 0.23	1.342 ± 0.00	2.371 ± 0.07	0.002 ± 0.01	0.005 ± 0.00
Gly	0.986 ± 0.17	1.135 ± 0.00	1.248 ± 0.02	0.001 ± 0.01	0.004 ± 0.00
Asp	2.013 ± 0.24	2.443 ± 0.08	2.134 ± 0.03	0.005 ± 0.01	0.010 ± 0.00
Glu	2.644 ± 0.06	2.648 ± 0.03	2.063 ± 0.19	0.008 ± 0.04	0.013 ± 0.00
Thr	0.873 ± 0.05	1.082 ± 0.01	0.751 ± 0.27	0.002 ± 0.00	0.004 ± 0.00
Ala	1.080 ± 0.02	1.365 ± 0.02	1.189 ± 0.07	0.002 ± 0.01	0.005 ± 0.00
Pro	0.913 ± 0.06	1.198 ± 0.01	1.533 ± 0.02	0.002 ± 0.00	0.004 ± 0.00
Cys	0.000 ± 0.00	0.128 ± 0.00	0.067 ± 0.05	0.000 ± 0.00	0.000 ± 0.00
Lys	1.115 ± 0.05	1.403 ± 0.03	1.092 ± 0.08	0.002 ± 0.00	0.005 ± 0.00
Tyr	0.743 ± 0.01	1.047 ± 0.01	0.668 ± 0.02	0.001 ± 0.00	0.003 ± 0.00
Met	0.309 ± 0.04	0.455 ± 0.00	0.321 ± 0.02	0.000 ± 0.00	0.001 ± 0.00
Val	0.980 ± 0.09	1.407 ± 0.01	1.056 ± 0.04	0.002 ± 0.00	0.004 ± 0.00
Ile	0.701 ± 0.11	1.321 ± 0.36	0.787 ± 0.03	0.001 ± 0.00	0.003 ± 0.00
Leu	1.568 ± 0.07	1.892 ± 0.34	1.812 ± 0.06	0.002 ± 0.00	0.007 ± 0.00
Phe	1.039 ± 0.02	1.411 ± 0.01	1.271 ± 0.01	0.001 ± 0.00	0.004 ± 0.00
Trp	0.127 ± 0.18	0.255 ± 0.00	0.000 ± 0.00	0.000 ± 0.01	0.000 ± 0.00

Means in the same column followed by different letters are statistically significant according to Tukey’s test at the level of *p* ≤ 0.05. Data are presented as mean ± SD (*n* = 3).

**Table 2:** Identified phytochemical compounds of broccoli fresh green leaves-originated green juice (GJ).

Identified Compounds	Formula	[M + H] + (*m*/*z*)	[M − H]^−^(*m*/*z*)
*Vitamins/vitamin like substances*			
Nicotinic acid	C_6_H_5_NO_2_	124.040	
Nicotinamide	C_6_H_6_N_2_O	123.056	
Cabagin-U (Vitamin U)	C_6_H_14_NO_2_S	164.075	
Pantothenic acid	C_9_H_17_NO_5_	220.119	
Riboflavin	C_17_H_20_N_4_O_6_	377.146	
Phylloquinone	C_31_H_46_O_2_	451.358	
Biotin	C_10_H_16_N_2_O_3_S	245.095	
*Flavonoids*			
*Chalcones*			
Isoliquiritigenin (2′,4,4′-trihydroxychalcone)	C_15_H_12_O_4_		255.065
*Flavonols*			
Quercetin (3,3′,4′,5,7-Pentahydroxyflavone)	C_15_H_10_O_7_		301.035
Quercetin-O-hexoside-O-hexosylhexoside isomer 1	C_33_H_40_O_22_		787.193
Quercetin-3-O-[caffeoyl-(→2)-glucosyl-(1→2)-glucoside]-7-O-glucoside	C_42_H_46_O_25_		949.225
Quercetin-O-(sinapoyl)hexosylhexoside-O-hexoside	C_44_H_50_O_26_		993.251
Quercetin-3-O-[feruloyl-(→2)-glucosyl-(1→2)-glucoside]-7-O-glucoside	C_43_H_48_O_25_		963.241
Quercetin-O-hexoside-O-hexosylhexoside isomer 2	C_33_H_40_O_22_		787.193
Quercetin-O-hexosylhexoside isomer 1	C_27_H_30_O_17_		625.140
Quercetin-di-O-hexoside	C_27_H_30_O_17_		625.140
Quercetin-O-hexosylhexoside isomer 2	C_27_H_30_O_17_		625.140
Quercetin-3-O-glucoside (Isoquercitrin)	C_21_H_20_O_12_		463.088
Kaempferol (3,4′,5,7-Tetrahydroxyflavone)	C_15_H_10_O_6_		285.040
Kaempferol-O-hexoside-O-hexosylhexoside	C_33_H_40_O_21_		771.198
Kaempferol-7-O-glucoside-3-O-sophoroside	C_3_3H_40_O_21_		771.198
Kaempferol-O-(caffeoyl)hexosylhexoside-O-hexoside	C_42_H_46_O_24_		933.251
Kaempferol-3-O-[caffeoyl-(→2)-glucosyl-(1→2)-glucoside]-7-O-glucoside	C_42_H_46_O_24_		933.251
Kaempferol-O-(caffeoyl)hexosylhexoside-O-hexosylhexoside	C_48_H_56_O_29_		1095.283
Kaempferol-3-O-[caffeoyl-(→2)-glucosyl-(1→2)-glucoside]-7-O-[glucosyl-(1→4)-glucoside]	C_48_H_56_O_29_		1095.283
Kaempferol-3-O-[sinapoyl-(→2)-glucosyl-(1→2)-glucoside]-7-O-glucoside	C_44_H_50_O_25_		977.256
Kaempferol-3-O-[sinapoyl-(→2)-glucosyl-(1→2)-glucoside]-7-O-[glucosyl-(1→4-)glucoside]	C_50_H_60_O_30_		1139.309
Kaempferol-3-O-[feruloyl-(→2)-glucosyl-(1→2)-glucoside]-7-O-glucoside	C_43_H_48_O_24_		947.246
Kaempferol-3-O-[feruloyl-(→2)-glucosyl-(1→2)-glucoside]-7-O-[glucosyl-(1→4)-glucoside]	C_49_H_58_O_29_		1109,299
Kaempferol-O-[p-coumaroyl-(→2)-glucosyl-(1→2)-glucoside]-7-O-glucoside	C_42_H_46_O_23_		917.235
Kaempferol-3,7-di-O-glucoside (Paeonoside)	C_27_H_30_O_16_		609,146
Kaempferol-O-(sinapoyl)hexosylhexoside-O-(sinapoyl)hexoside	C_55_H_60_O_29_		1183.314
Kaempferol-di-O-hexoside	C_27_H_30_O_16_		609.146
Kaempferol-O-(caffeoyl)hexosylhexoside	C_36_H_36_O_19_		771.177
Kaempferol-O-(sinapoyl)hexosylhexoside	C_38_H_40_O_20_		815.203
Kaempferol-7-O-sophoroside	C_27_H_30_O_16_		609.146
Kaempferol-O-(feruloyl)hexosylhexoside	C_37_H_38_O_19_		785.193
Kaempferol-O-(4-coumaroyl)hexosylhexoside	C_36_H_36_O_18_		755.182
Kaempferol-O-(disinapoyl)hexosylhexosylhexoside-O-hexoside	C_61_H_70_O_34_		1345.367
Kaempferol-O-hexosylhexoside	C_27_H_30_O_16_		609.146
Kaempferol-3-O-glucoside (Astragalin)	C_21_H_20_O_11_		447.093
Isorhamnetin-O-hexosylhexoside	C_28_H_32_O_17_		639.156
Isorhamnetin-3-O-glucoside	C_22_H_22_O_12_		477.103
Isorhamnetin-7-O-glucoside-3-O-sophoroside (Brassicoside)	C_34_H_42_O_22_		801.209
*Flavanones*			
4′.7-Dihydroxyflavanone (Liquiritigenin)	C_15_H_12_O_4_		255.066
4′,5,7-Trihydroxyflavanone (Naringenin)	C_15_H_12_O_5_		271.061
*Flavons*			
Apigenin (4′,5,7-Trihydroxyflavone)	C_15_H_10_O_5_		269.045
Apigenin-7-O-glucuronide	C_21_H_28_O_11_		445.077
Luteolin (3′.4′.5.7-Tetrahydroxyflavone)	C_15_H_10_O_6_		285.039
*Phenolic acids*			
Neochlorogenic acid (5-O-Caffeoylquinic acid)	C_16_H_18_O_9_	355.103	
Chlorogenic acid (3-O-Caffeoylquinic acid)	C_16_H_18_O_9_	355.103	
Chryptochlorogenic acid (4-O-Caffeoylquinic acid)	C_16_H_18_O_9_	355.103	
Caffeic acid	C_9_H_8_O_4_		179.034
4-Coumaric acid	C_9_H_8_O_3_		163.040
Sinapic acid	C_11_H_12_O_5_	225.076	
Di-O-sinapoylgentiobiose	C_34_H_42_O_19_		753.224
Tri-O-sinapoylgentiobiose	C_45_H_52_O_23_		959.282
Feruloyl-sinapoyldihexoside	C_33_H_40_O_18_		723.214
Di-O-sinapoylglucose	C_28_H_32_O_14_		591.171
Feruloyl-disinapoyldihexoside	C_44_H_50_O_22_		929.272
Syringaldehyde (3,5-Dimethoxy-4-hydroxybenzaldehyde)	C_9_H_10_O_4_	183.066	
*Glucosinolates*			
Glucobrassicin (3-Indolylmethyl glucosinolate)	C_16_H_20_N_2_O_9_S_2_	447.053	
3-Methylsulphinylpropyl isothiocyanate	C_5_H_9_NOS_2_	164.020	
4-Methoxy-3-indolylmethyl glucosinolate	C_17_H_22_N_2_O_10_S_2_	477.064	
Sulforaphane	C_6_H_11_NOS_2_	178.036	
Neoglucobrassicin (1-Methoxy-3-indolylmethyl glucosinolate)	C_17_H_22_N_2_O_10_S_2_	477.064	
*Coumarins*			
Scopoletin (7-Hydroxy-6-methoxycoumarin)	C_10_H_8_O_4_	193.050	
*Other phytocompounds*			
γ-Aminobutyric acid	C_4_H_9_NO_2_	104.071	
Indole-4-carbaldehyde	C_9_H_7_NO	146.061	
Abscisic acid	C_15_H_20_O_4_		263.128
Kynurenic acid	C_10_H_7_NO_3_	190.050	

**Table 3:** Quantitative alterations of vitamins in broccoli green leaves-derived products by different fractionation processes (*Process 1; Process2 and Process 3*): freeze-dried green juice (GJ); leaf protein concentrate obtained by microwave coagulation (MW-LPC); leaf protein concentrate obtained by lactic acid fermentation (LA-LPC); brown juice obtained by microwave coagulation (MW-BJ); and brown juice obtained by lacto-fermentation (LA-BJ).

Vitamins	GJ	MW-LPC	LA-LPC	MW-BJ	LA-BJ
	μg g^−1^ DW			ng mL^−1^	
Nicotinamide	9.71 ± 0.17 ^a^	6.95 ±0.17 ^b^	4.03 ± 0.20 ^c^	839.73 ± 101.00 ^a^	386.36 ± 16.77 ^b^
Nicotinic acid	19.18 ± 0.27 ^a^	11.97 ± 0.14 ^b^	11.33 ± 0.43 ^b^	2093.35 ± 480.15 ^a^	1620.00 ± 10 ^a^
Biotin	1.54 ± 0.06 ^a^	nd ^‡^	nd	nd	nd
Riboflavin	5.01 ± 0.11 ^b^	3.85 ± 0.08 ^c^	5.35 ± 0.03 ^a^	644.37 ± 19.85 ^b^	1069.55 ± 5.46 ^a^

^‡^ not detected. Means in the same column followed by different letters are statistically significant according to Tukey’s test at the level of *p* ≤ 0.05. Data are presented as mean ± SD (*n* = 3).

**Table 4:** Quantitative changes of flavonoids in broccoli green leaves-originated products by different processes (*Process 1; Process2 and Process 3*): freeze-dried green juice (GJ); leaf protein concentrate obtained by microwave coagulation (MW-LPC); leaf protein concentrate obtained by lactic acid fermentation (LA-LPC); brown juice obtained by microwave coagulation (MW-BJ); and brown juice obtained by lacto-fermentation (LA-BJ).

Flavonoids	GJ	MW-LPC	LA-LPC	MW-BJ	LA-BJ
	μg g^−1^ DW			ng mL^−1^	
Isoliquiritigenin (2′,4,4′-trihydroxychalcone)	0.22 ± 0.03 ^a^	0.24 ± 0.04 ^a^	0.21 ± 0.03 ^a^	1.57 ± 0.09 ^b^	2.10 ± 0.07 ^a^
Quercetin (3,3′,4′,5,7-Pentahydroxyflavone)	1.90 ± 0.24 ^c^	20.26 ± 0,53 ^b^	48.75 ± 1.30 ^a^	209.66 ± 116.18^a^	7390.00 ± 275.13 ^b^
Kaempferol (3,4′,5,7-Tetrahydroxyflavone)	2.63 ± 0.12 ^c^	20.91 ± 0.30 ^b^	895.26 ± 17.13^a^	340.00 ± 45.83 ^b^	13046.67 ± 1956.76 ^a^
4′.7-Dihydroxyflavanone (Liquiritigenin)	0.14 ± 0.02 ^a^	0.14 ± 0.01 ^a^	0.13 ± 0.02 ^a^	1.19 ± 0.06 ^b^	1.37 ± 0.04 ^a^
Naringenin (4′,5,7-Trihydroxyflavanone)	1.08 ± 0.06 ^b^	1.08 ± 0.05 ^b^	3.13 ± 0.14 ^a^	nd	38.18 ± 1.85 ^a^
Genkwanin	0.12 ± 0.01 ^a^	nd ^‡^	0.14 ± 0.02 ^a^	nd	14.73 ± 0.32 ^a^
Luteolin	0.25 ± 0.03 ^b^	0.26 ± 0.03 ^b^	2.04 ± 0.11 ^a^	23.79 ± 9.47 ^b^	80.34 ± 3.32 ^a^
Apigenin	0.15 ± 0.02 ^ab^	0.11 ± 0.02 ^b^	0.20 ± 0.02 ^a^	1.85 ± 0.16 ^a^	2.04 ± 0.10 ^a^
Apigenin-7-O-glucuronide	0.17 ± 0.02 ^ab^	0.14 ± 0.02 ^b^	0.22 ± 0.02 ^a^	1.09 ± 0.04 ^b^	1.17 ± 0.04 ^a^

^‡^ not detected. Means in the same column followed by different letters are statistically significant according to Tukey’s test at the level of *p* ≤ 0.05. Data are presented as mean ± SD (*n* = 3).

**Table 5:** Quantitative changes of phenolic acids/glucosinolates in broccoli green leaves-derived products by different processes (*Process 1; Process2 and Process 3*): freeze-dried green juice (GJ); leaf protein concentrate obtained by microwave coagulation (MW-LPC); leaf protein concentrate obtained by lactic acid fermentation (LA-LPC); brown juice obtained by microwave coagulation (MW-BJ); and brown juice obtained by lacto-fermentation (LA-BJ).

	GJ	MW-LPC	LA-LPC	MW-BJ	LA-BJ
Phenolic Acids/Glucosinolates	μg g^−1^ DW			ng mL^−1^	
Chlorogenic acid (3-O-Caffeoylquinic acid)	26.36 ± 2.02 ^a^	26.52 ± 0.47 ^a^	n.d	3723.33 ± 183.39 ^b^	32.36 ± 4.68 ^a^
Neochlorogenic acid (5-O-Caffeoylquinic acid)	644.71 ± 16.12 ^a^	530.81 ± 22.51 ^b^	n.d	94880.00 ± 4762.02 ^a^	4.94 ± 1.20 ^b^
Cryptochlorogenic acid (4-O-Caffeoylquinic acid)	57.15 ± 2.20 ^b^	76.18 ± 2.08 ^a^	n.d	16733.33 ± 1095.10 ^a^	12.13 ± 0.56 ^b^
Syringaldehyde	0.39 ± 0.03 ^b^	1.27 ± 0.03 ^a^	0.17 ± 0.03 ^c^	301.12 ± 81.34 ^a^	34.46 ± 3.59 ^b^
p-Coumaric acid	2.73 ± 0.20 ^b^	8.26 ± 0.15 ^a^	0.23 ± 0.05 ^c^	2623.33 ± 1114.64 ^b^	22.92 ± 0.75 ^a^
Ferulic acid	4.71 ± 0.21 ^b^	11.51 ± 0.48 ^a^	3.55 ± 0.25 ^c^	6923.33 ± 3122.38 ^b^	530.29 ± 32.98 ^a^
Sinapic acid	352.20 ± 10.60 ^a^	208.51 ± 8.19 ^b^	109.09 ± 11.91 ^c^	20190.00 ± 1047.62 ^a^	8376.67 ± 370.04 ^b^
Sulforaphane	17.65 ± 0.12 ^a^	12.57 ± 0.25 ^b^	n.d	2083.33 ± 456.33 ^a^	n.d

Means in the same column followed by different letters are statistically significant according to Tukey’s test at the level of *p* ≤ 0.05. Data are presented as mean ± SD (*n* = 3).

## Data Availability

Data is contained within the article.
